# A transnational perspective of global and regional ecosystem service flows from and to mountain regions

**DOI:** 10.1038/s41598-019-43229-z

**Published:** 2019-04-30

**Authors:** Uta Schirpke, Ulrike Tappeiner, Erich Tasser

**Affiliations:** 1 Department of Ecology, University of Innsbruck, Sternwartestrasse 15, 6020 Innsbruck, Austria; 2 Institute for Alpine Environment, Eurac Research, Viale Druso 1, 39100 Bozen/Bolzano, Italy

**Keywords:** Ecosystem services, Environmental social sciences

## Abstract

The spatial relationships of ecosystem services are complex and poorly understood due to spatial mismatches between areas of provision and the areas that benefit. In this study, we assess the spatial flows of six key ecosystem services from and to mountain regions at the regional and global level. We identify major directions of spatial flow and illustrate different types and transfer mechanisms with detailed examples focusing on the European Alps and surrounding lowlands. Our results demonstrate that the spatial flows of ecosystem services range from local to global interactions and extend far beyond the regional level for most of the ecosystem services assessed. Transportation processes encompass passive biophysical processes and the active transportation of goods, distribution of information and traveling of people. Decision and policy-making can use this enhanced understanding to influence ecosystem service transfer and consequently manage natural resources in a sustainable way.

## Introduction

The importance of integrating ecosystem services into landscape management, decision-making and policy development is widely acknowledged as fostering the sustainable use of natural resources^1,2^. Research on general definitions, concepts and frameworks to assess ecosystem services has advanced rapidly in recent decades, but the operational implementation of the concept of ecosystem services into decision-making and the management of natural resources lags behind^3,4^. One reason pertains to an insufficient understanding of spatial relationships among ecosystem services, as areas providing specific ecosystem services are often widely dislocated from those that benefit^5^. For example, mountain regions often represent important water suppliers for people living in large cities in the adjacent lowland areas or for people who use nearby green spaces for recreational activities. Products from agriculture are traded worldwide and the sequestration of greenhouse gas emissions is of global relevance. Therefore, in order to benefit from ecosystem services, it is necessary to facilitate transfer from the supply area to the receiving area either by transporting goods to the beneficiaries or by requiring people to move to the area where a certain ecosystem service is provided^6,7^. Therefore, a detailed understanding of the different types of movement is crucial to adequately managing ecosystem services not only at the local or regional level but also at the cross-national or global level^8^.

The spatial relationships or flows of ecosystem services have been conceptualized by several studies, contingent on the spatial distribution of the supply and demand of a specific ecosystem service. These frameworks describe different types, directions and spatial scales of relationships, as some ecosystem services are consumed *in situ*, others follow specific directions (mountain-lowland, coast-inland), and still others are even related to global distribution^9^. The general scheme based on providing and benefiting areas has been extended by introducing connecting areas, which are necessary to overcoming spatial mismatches between the former two types of area^5,10^. The spatial routing of ecosystem services, i.e., the transfer from provisioning areas to locations of use, has also been conceptualized in quantitative frameworks at different spatial scales^8,11–14^. Such modelling of the routing requires a huge amount of data and therefore tends to concentrate on the local scale^6^, whereas regional or global studies remain focused on a rather theoretical level or on the potential provision of ecosystem services as opposed to their actual use^15,16^. Spatial relationships additionally depend on the socio-ecological system, as human interactions influence the level of ecosystem services provision through land management decisions, and different societies may value and demand specific ecosystem services in different ways^17^. Specifically, studies have addressed flows of ecosystem services along rural-urban gradients^18,19^, from protected areas to the adjacent beneficiaries^20,21^ or within watersheds^22^, but global ecosystem services flows from mountain regions have only been analysed in terms of spatial mismatches^23^.

This study therefore aims to analyse the spatial flows of ecosystem services from the European Alps at the regional to global level. The term ‘ecosystem service flow’ may refer to actual service provision^24,25^ or to the transfer path from supply to demand areas^11^. Here we use the term in the latter sense, i.e., the transfer of ecosystem services between supply and demand areas. For each ecosystem service we identify major directions of spatial flow, and illustrate the different types and transfer mechanisms with detailed examples based on the mapping and quantification of supply, demand, and actual use.

## Results

**Figure 1:**
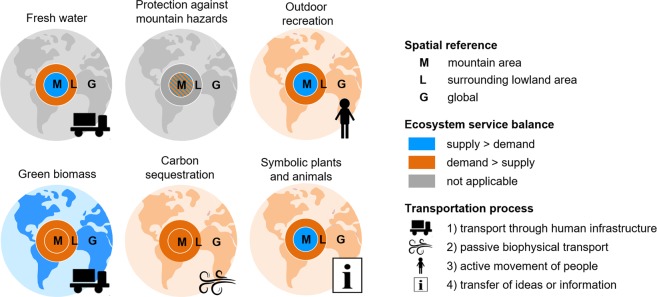
Generalized schemes of ecosystem services transfer for six key ecosystem services of mountain regions. For each spatial reference (M, L, G), the colours indicate whether this area is a service-providing (supply > demand) or a service-demanding area (demand > supply). The different types of transportation processes (1–4) are represented by different symbols.

Following several theoretical frameworks on the spatial characteristics of ecosystem services transfer^5,8–10,12^, we identified general types of ecosystem service flow at the regional and global level typical of mountain regions (Fig. 1). We refer to three spatial reference levels including mountains (M), surrounding lowland areas (L) and global (G). We then classified these spatial areas as mainly service-providing areas (supply > demand) or predominantly service-demanding areas (demand > supply)^5^, in case they are included in the flow of ecosystem services from or to mountain regions. The transportation processes included several types: (1) transfer of goods through a human-made carrier from supply areas to demand areas^5,8^, which is largely independent from landscape structures as goods are transported to the consumer using human infrastructure such as roads, railroads, shipping and aviation routes, and pipelines^6^; (2) movement of people to benefit from a specific ecosystem service, depending on accessibility^5^; (3) passive biophysical flow through ecological processes, e.g., from polluting areas to service-providing ecosystems^8^; and (4) transfer of ideas or information through human-made communication channels^8^. Furthermore, there exist local services that either concentrate on mountain or lowland areas not requiring any transfer^5^.

### Fresh water

**Figure 2:**
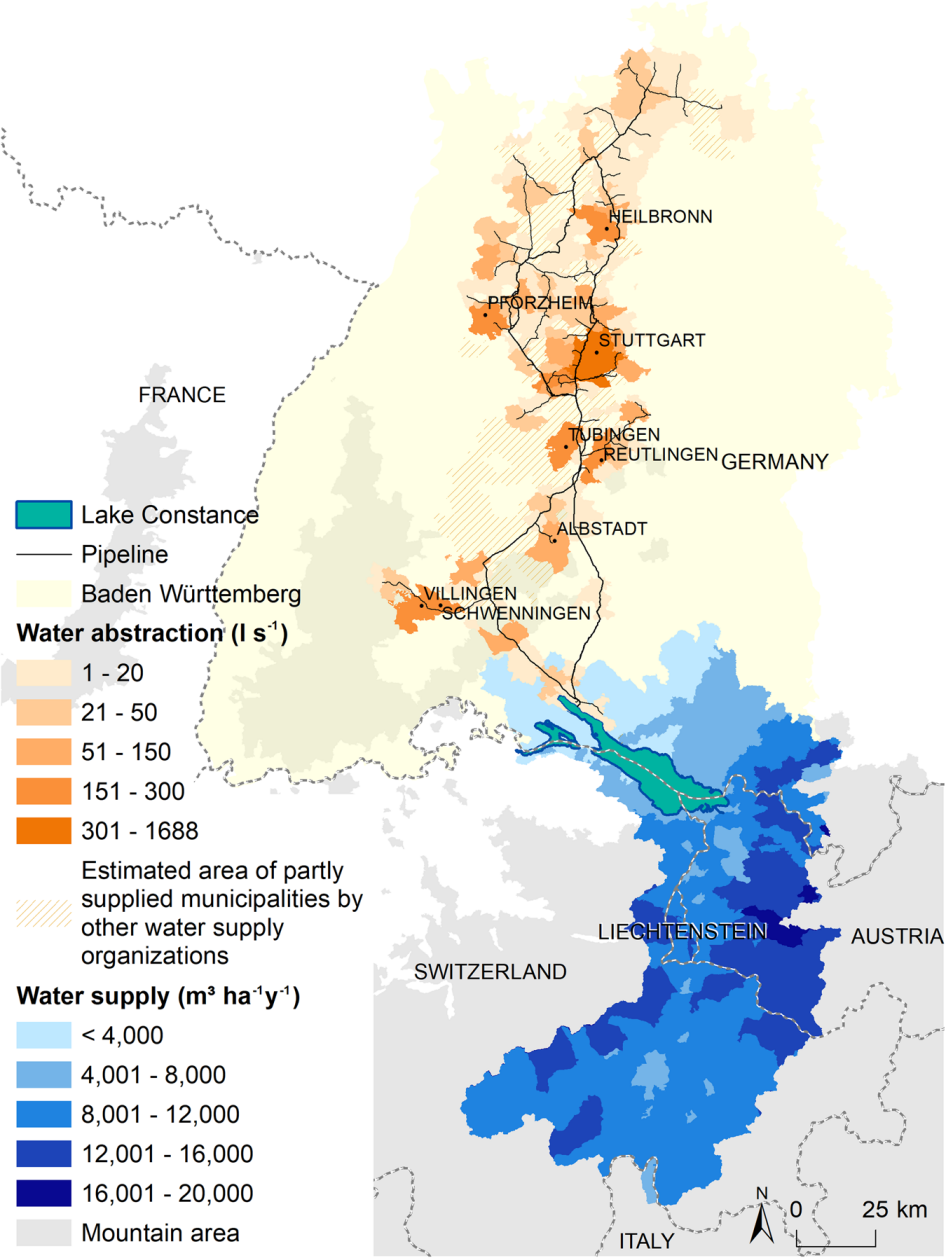
Transfer of fresh water through pipelines from Lake Constance to consumers located in Baden-Württemberg, Germany. Lake Constance is mainly supplied by mountain areas. In addition to the municipalities indicated, water from Lake Constance is distributed to further locations via other water supply organizations.

The distribution of fresh water usually requires pipelines to transport it from supply areas to consumers’ homes, and the major direction of flow is from the mountains to the lowlands. In our case study, Lake Constance primarily receives rainwater and meltwater from the Alps (78% of the total amount of water), which is then transported by the River Rhine (Fig. 2). About 11.5 billion cubic meters of water pass through Lake Constance annually, of which 125 million cubic meters are abstracted for drinking water of the Lake Constance Water Supply (https://www.bodensee-wasserversorgung.de). This corresponds to almost one third of global consumption of bottled water in 2017^26^. The water supplier distributes the water to approximately 4 million people in Baden-Württemberg living in 320 cities and municipalities. The water is delivered via a pipeline system of 1,700 km length and takes up to seven days from its extraction point at Lake Constance to the users in northern Baden-Württemberg. The economic value of drinking water amounts to 269 million € per year.

### Grassland biomass

**Figure 3:**
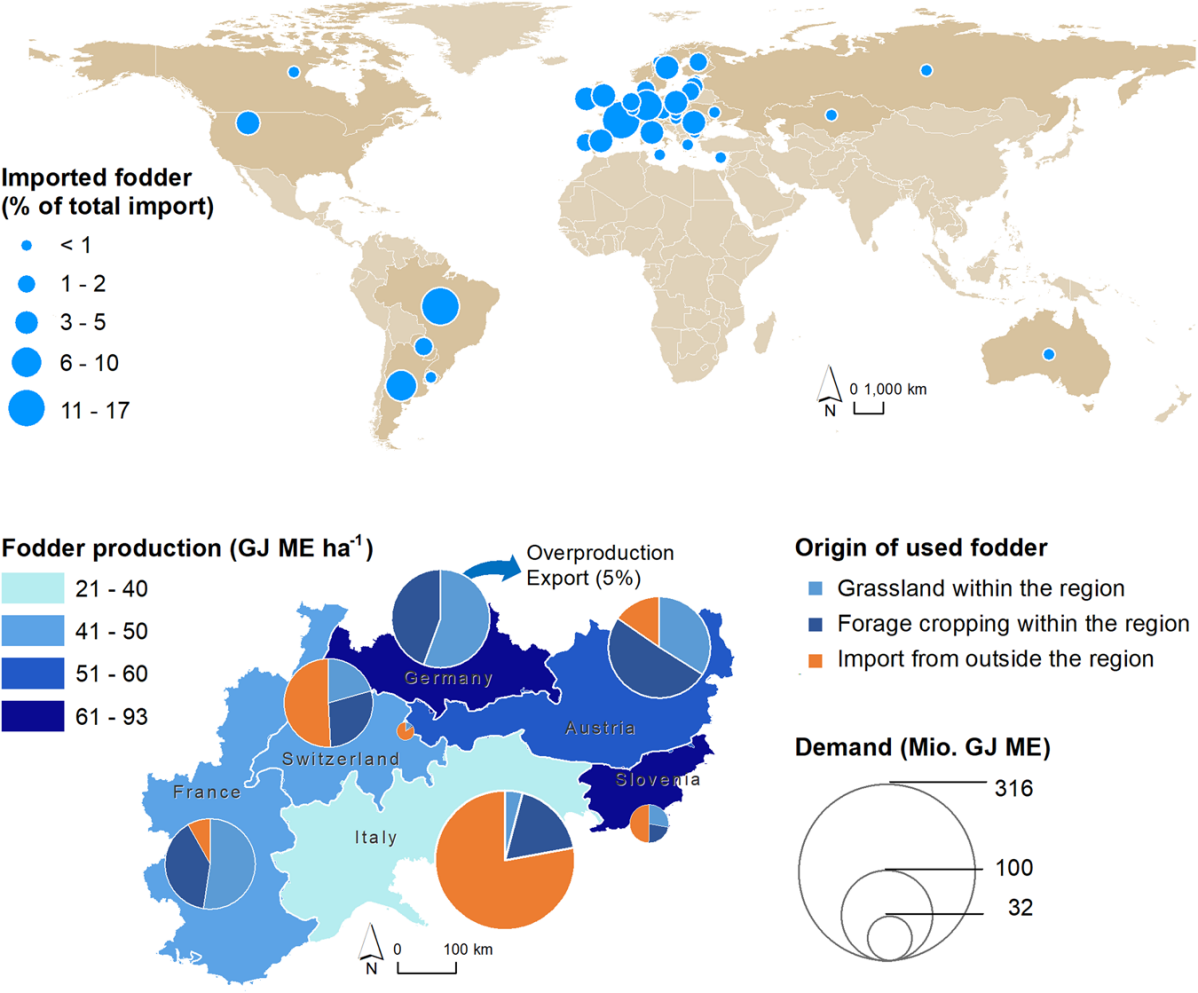
Most important countries for imported concentrated feeds to the European Alps (above). Fodder production and demanded fodder for each country within the Alpine Space area, distinguishing the different sources of fodder (below).

A significant fraction of the demand in the Alps and their lowlands was not met by the supply of forage from pastures and meadows. The demand was integrated by inputs from crop farming and imports of concentrated feeds. The highest share of concentrated feeds originated from European Union (EU) countries (70%), yet 23% of the total imported fodder came mainly from countries in South America (Fig. 3). While Switzerland, Slovenia and the Italian regions imported more than 50% of the fodder actually required, the German part of the study area produced even more fodder than the demanded and exported 5% of the produced fodder to other regions. The economic value of the total amount of produced fodder was estimated to 6.5 billion € per year, while the imported fodder equals 5.1 billion € per year (information for each country is provided in Supplementary Table 1).

### Protection against mountain hazards

**Figure 4:**
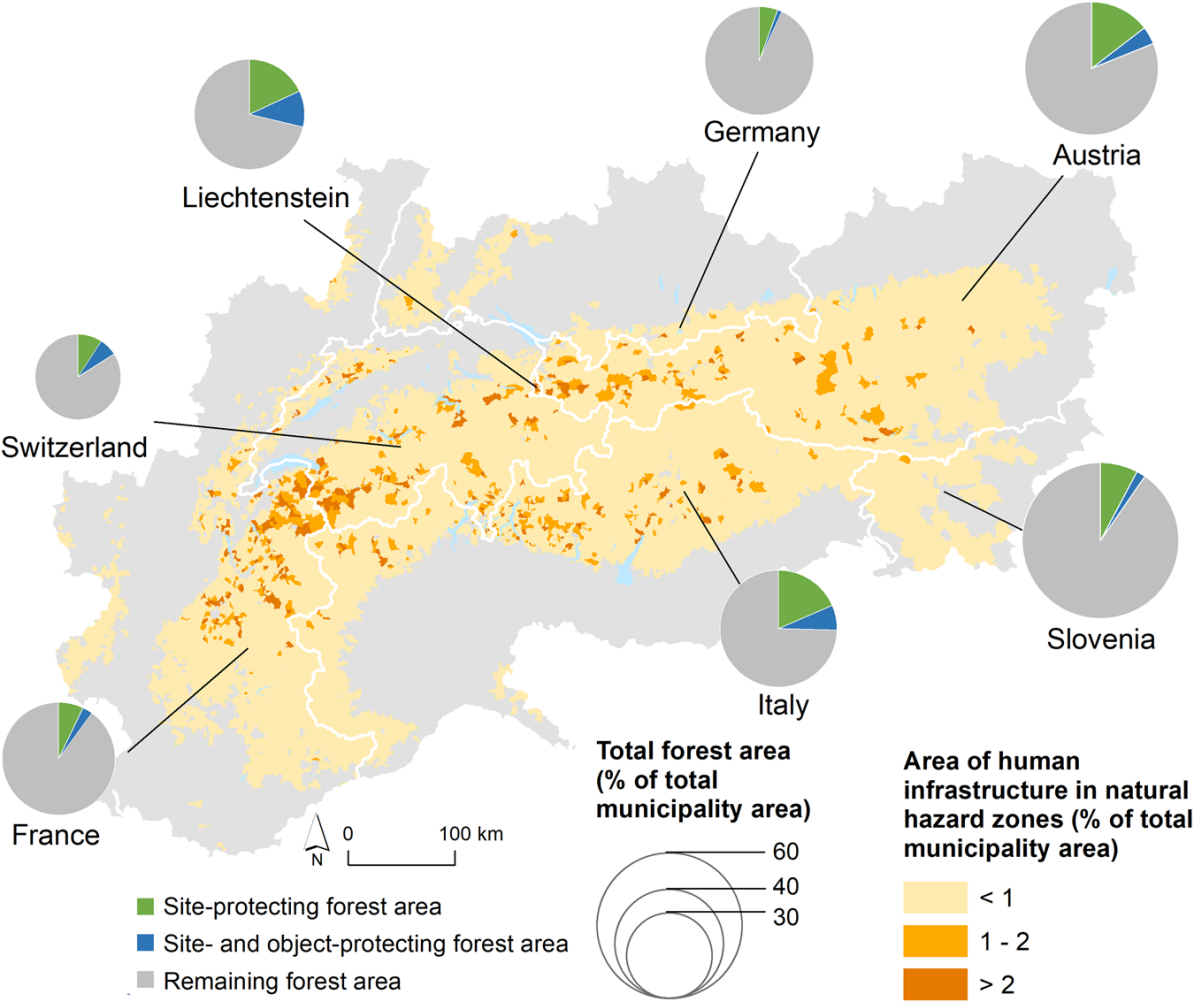
Demand for protection against mountain hazards in mountain municipalities and share of forest actually protecting human settlements and infrastructure against mountain hazards (object-protecting forest) as part of the site-protecting forest compared to the total forest area.

This ecosystem service was limited in selected locations in mountain areas (Fig. 4). With over 25%, Italy and Liechtenstein possessed the highest proportion of site-protecting forest area of the total forest area, while Germany, France and Slovenia were covered by no more than 10% of site-protecting forest (Supplementary Table 3). Approximately 40% of this site-protecting forest area served to protect human settlements and infrastructure (object-protecting forest) in Liechtenstein and Switzerland, but only about 20% in Austria, Germany and Slovenia. The economic value of the object-protecting forest, estimated by the replacement cost method, amounted to 5.2 billion € yearly, in the case that the total forest cover would be removed and its natural protecting function would not be longer available.

### Carbon sequestration

**Figure 5:**
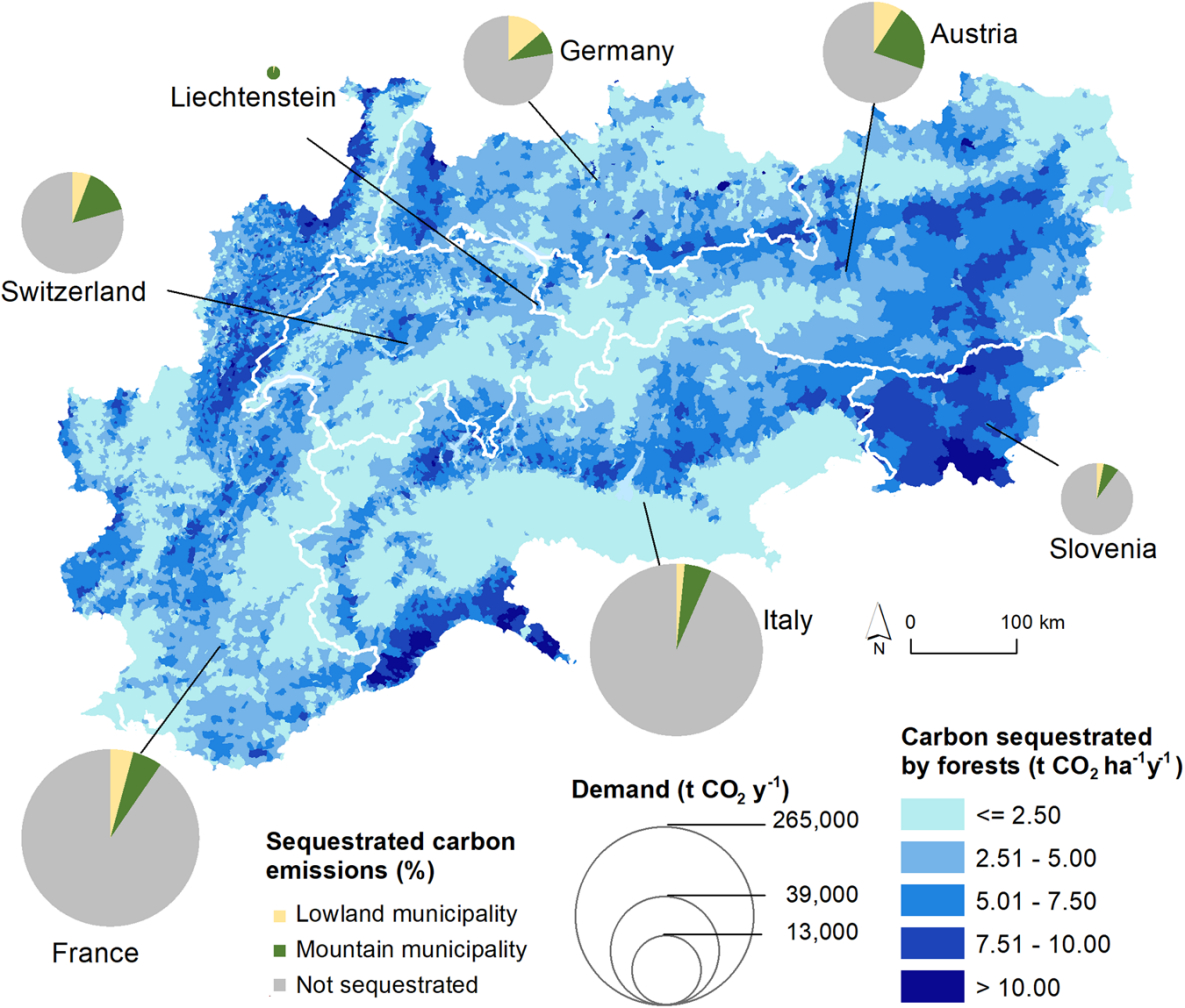
Carbon sequestrated by forests at the municipality level. Supply and demand are summarized for each country, distinguishing lowland and mountain areas. The proportion of “not sequestrated” carbon emissions indicates the remaining demand at the national level.

In all countries, regional demand greatly exceeded the rates of carbon sequestration provided by forests (Fig. 5, Supplementary Table 5). Mountain areas in the study area could generally sequestrate greater amounts (22% of the demand within mountain municipalities) than lowland areas (6% of the demand in lowland municipalities). The total amount of sequestrated carbon in the study area corresponded to almost 1 million € (values for each country are reported in Supplementary Table 5). However, 89% of the carbon emissions in the entire study area could not be sequestrated, ranging from 70% in Austria to 93% in Italy.

### Outdoor recreation

**Figure 6:**
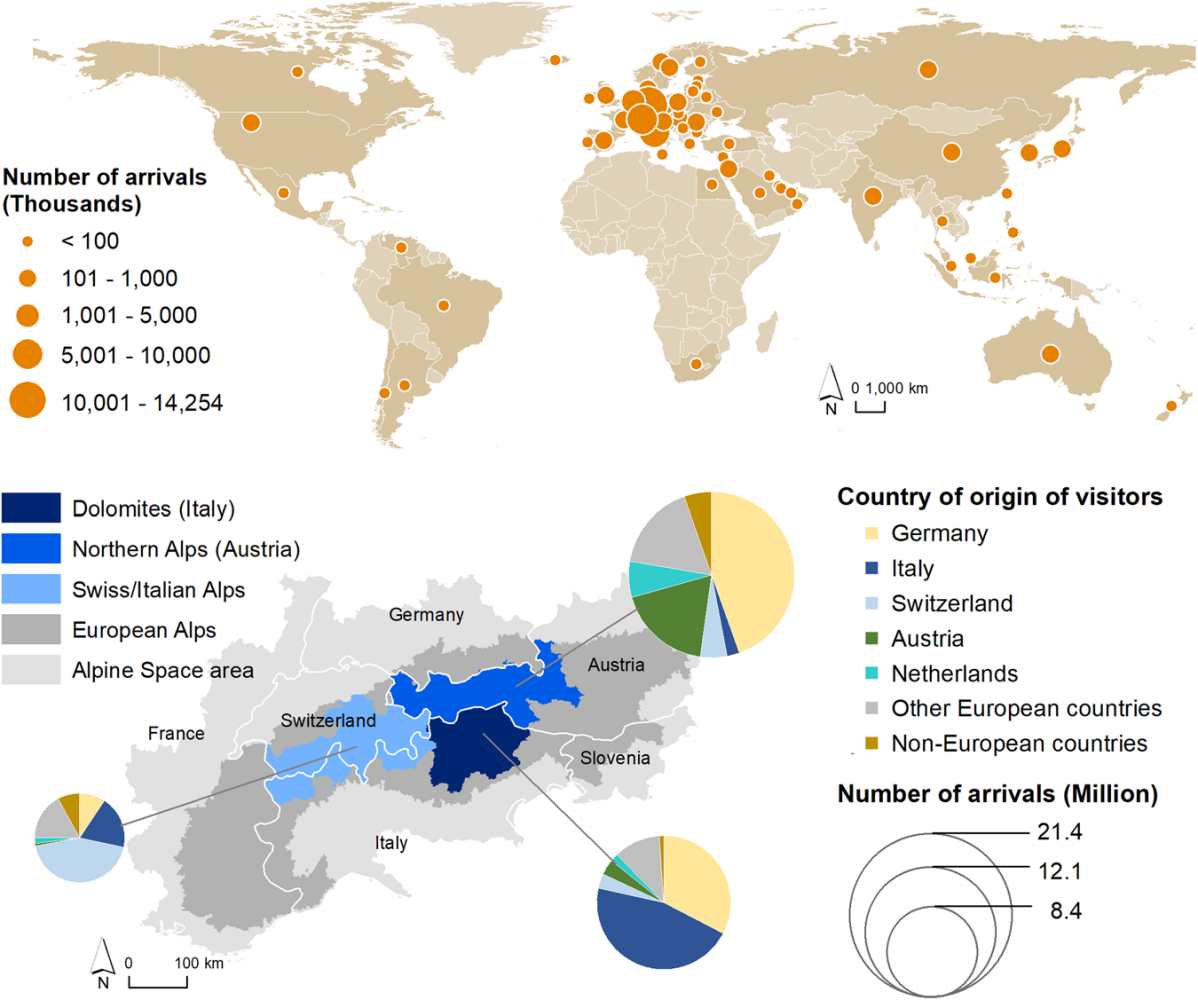
Most important countries of origin of visitors to major hotspots of outdoor recreation in the European Alps (above). Distribution of visitors in 2017 to three analysed hotspot zones and related countries of origin of the visitors (below).

People from all over the world visited destinations in the European Alps (Fig. 6, Supplementary Tables 6 and 7). Regarding the example of three significant tourism destinations, we demonstrate important visitor flows for the European Alps. Most visitors (95%) came from European countries, especially Germany (34%), Italy (18%), Switzerland (12%) and Austria (11%). Whereas in the selected areas in Switzerland and Italy about half of the visitors came from their own country, the Northern Alps in Austria were predominantly visited by people from foreign countries (82%). The visitors contributed with 16.1 billion € to the local economies of the three hotspots in the year 2017 (for further details, see Supplementary Table 7).

### Symbolic plants and animals

**Figure 7:**
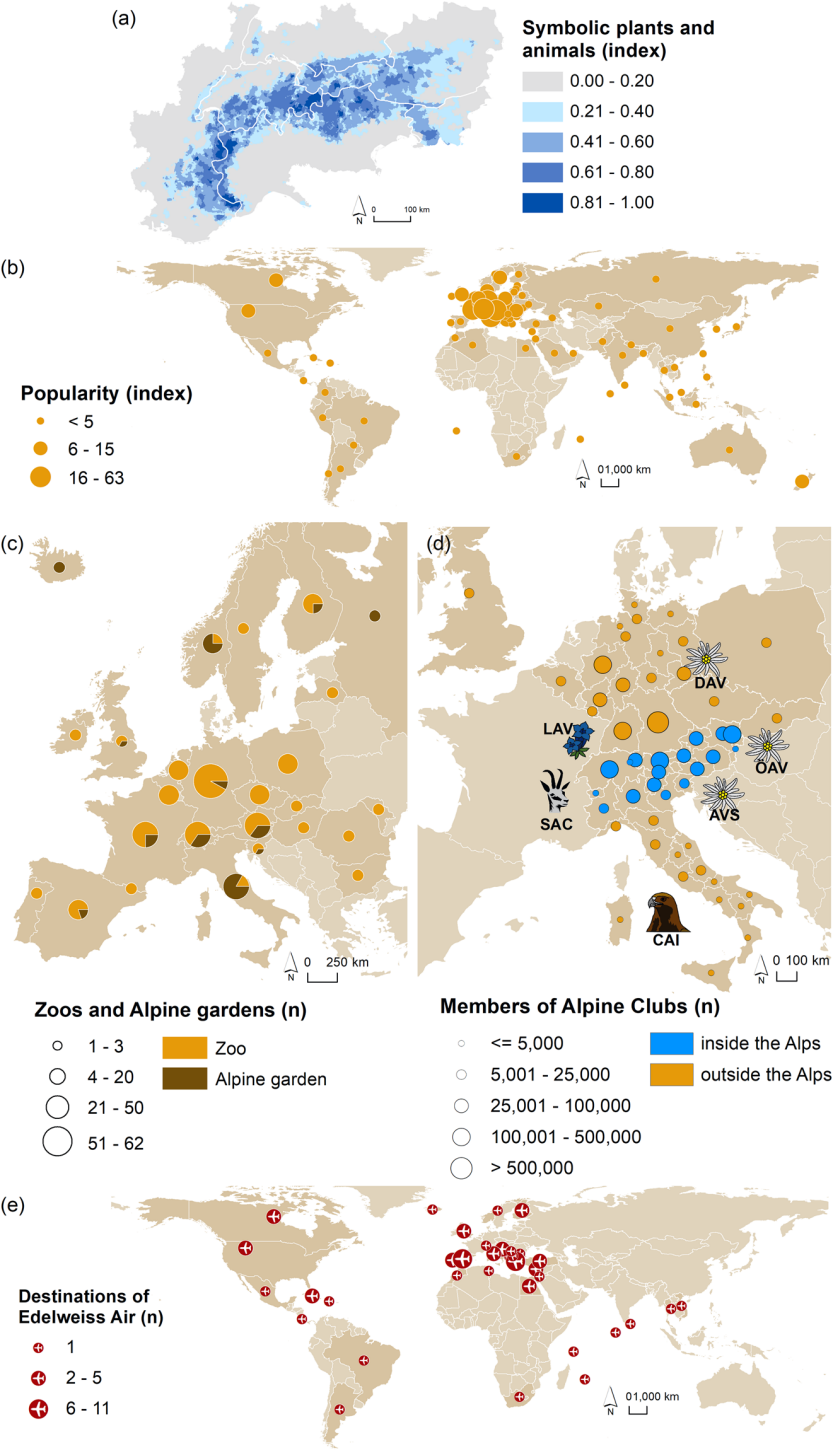
(**a**) Spatial distribution of the supply of symbolic plants and animals (adapted from Schirpke *et al*.^45^). (**b**) Popularity of search terms derived from Google Trends (https://www.google.com/trends) of symbolic plants and animals and (**c**) occurrence of symbolic plants and animals in zoos and botanical gardens in Europe, indicating the demand. (**d**) Spatial distribution of members of Alpine Clubs of different Alpine countries. The logos of these Alpine Clubs refer to plants or animals that are symbolic for the European Alps: the German (DAV), Austrian (ÖAV) and South Tyrol (AVS) Alpine Clubs are represented by the edelweiss, the Swiss Alpine Club (SAC) by the chamois, the Italian Alpine Club (CAI) by the eagle, and the Liechtenstein Alpine Club (LAV) by the gentian. Symbols in the map are only used for illustrative purposes and do not represent the logos of the Alpine Clubs. (**e**) Destinations of Edelweiss Air carrying the edelweiss as a symbol for the Alps, in specific for Switzerland.

Symbolic plants and animals occur mainly in high elevated areas of the European Alps (Fig. 7a). The interest in symbolic plants and animals is reflected in the search interest of people using the internet. The results from Google Trends (https://www.google.com/trends) indicate a high popularity in European countries, especially in Italy (popularity index 63), Switzerland (popularity index 55), Slovenia (popularity index 49), and Austria (popularity index 38); further details in Supplementary Table 9. Accordingly, the existence of such symbolic animals in 168 zoos all over Europe (of which 25% located in the Alps) suggests that a certain demand exists to observe these animals in and outside the Alps (Fig. 7c). Similarly, Alpine plants can be found in botanical gardens, of which 65% are located in the Alps.

Plants and animals are used in manifold ways to convey information and ideas from Alpine regions also to locations outside the Alps. One type of continuous transfer of ideas and information occurs from the Alps to people living beyond the mountains through Alpine Clubs, as members of Alpine clubs regularly receive information on activities and Alpine locations via journals, newsletters, meetings, and so forth. Alpine clubs that refer to symbolic plants or animals in their logos included 2.4 million members in 2017 distributed over several states (Fig. 7d). In countries located completely or almost completely within the European Alps, the percentage of members was higher (e.g., South Tyrol 14.5%, Liechtenstein 7.4% and Austria 5.9%) than in those only partly included within the Alps (e.g., Switzerland 1.8%, Germany 1.6% and Italy 0.5%). Nevertheless, people living far from the Alps were still members of the Alpine clubs, organized in sub-groups but carrying the same symbolic plants or animals in their logos. Another example is Edelweiss Air, which in 2018 carried 2.4 million passengers (https://www.flyedelweiss.com/) to almost 70 destinations worldwide (Fig. 7e). The naming and the logo of the airline transferred at the same time a specific image and idea related the Alps to these destinations.

## Discussion

Numerous studies have demonstrated that areas of either high supply or demand regarding service-provisioning and -benefiting areas are significantly dislocated due to spatial divergence between natural or semi-natural ecosystems and human-dominated environments^7,10,20,21,25^. In our case, this means that more natural mountain regions are hotspots of ecosystem services supply^23^, whereas high demand is mostly associated with highly urbanized areas or intensively used agricultural areas in the lowlands^18^. Consequently, actual use may depend on the spatial distribution of supply or demand to varying degrees, but it may also be completely independent^27^.

The transfer of ecosystem services between these different areas includes various types of transportation processes, ranging from passive biophysical processes to the active transport of goods or traveling of people. Our examples also indicate the major direction of flow from mountain areas to lowlands, as well as global interactions that extend far beyond the regional level. The only service restricted to selected mountain areas is protection against mountain hazards, whereas all other ecosystem services are interwoven with the global trading of goods in the case of provisioning services or the demand for people’s cultural services. Therefore, our examples capture certain aspects, although further wide-ranging dependencies of lowland regions on mountain areas need to be considered^23^. For example, we have illustrated that about 4 million people receive fresh water captured from only 1% of Lake Constance’s water reservoir, but all downstream regions benefit from the above-average water supply of the Alpine catchment of the River Rhine^28^. The importance of the Alps as a water supply is linked to high precipitation rates, low evapotranspiration, and delayed runoff due to temporarily stored water as snow and ice^29^.

A second very important service is represented by recreational opportunities, and specifically what mountain environments offer to local people and visitors^30^. Here we exemplified the flow of tourists to locations that primarily offer outdoor activities, while surrounding cities such as Bern, Zurich, Munich and Milan host even more international tourists who may also visit nearby mountain locations. Nevertheless, our results demonstrate high levels of global interactions, as people from all over the world benefit from the recreational services offered by mountain regions, confirming the complex spatial socio-ecological relationships of cultural services^31^. The actual use of outdoor recreation is thus influenced both by natural assets and proximity to benefiting areas in the case of green urban areas^30^. Moreover, it also depends on tourism infrastructure and the promotion and popularity of destinations^32,33^.

Our analyses concentrated on a quantitative analysis of the transfer of ecosystem services, but the monetary valuation of ecosystem services is often applied to support policy and decision-making, as this is an effective way to compare costs and benefits^1,2^. In our study, we estimated economic values for all ecosystem services based on established valuation methods such as market-price based approaches or the replacement cost method, with the exception of ‘symbolic plants and animals’. For this ecosystem service, we could not relate the transfer of information or ideas to monetary values, as the influence of the used logos on the number of members or passengers was not identifiable. While for most provisioning services and some regulating services real markets exist, especially for cultural ecosystem services, an economic valuation is rather critical, because the obtained values are often limited to a certain aspect of the ecosystem service in question and greatly depend on the applied valuation method^34^. Such results may therefore not easy to understand by stakeholders and decision makers^4^. Moreover, an economic value cannot sufficiently reveal the ecological and social importance of ecosystem services; non-monetary valuation methods, assessing human preferences and values, may be more suitable to assess socio-ecological values related to ecosystem services^35^.

Our study demonstrates the tight spatial connections and dependencies of people on mountain areas in the provision of ecosystem services at different spatial levels. To foster the sustainable use of natural resources in and beyond the mountains, efficient planning strategies must account for wide-ranging and complex spatial relationships between areas of supply and areas of demand. Given that most transportation schemes require human interaction, especially in order to transport goods to consumers or to enable the movement of people to benefit from specific services, decision-makers and policy-makers can use this enhanced understanding to influence ecosystem service transfer and consequently manage natural resources in a sustainable way. Here, we focused on the European Alps, but we recommend to carry out similar analyses for other mountain regions in the world, e.g., the Andes, the Carpathians, the Himalayas, and the Rocky Mountains. In particular such analysis can provide valuable information to conservation policies in less protected mountain regions such as the Mexican and Central American highlands or the Drakensbergs.

## Materials and Methods

### Key ecosystem services in the European Alps

In order to analyse spatial relationships between mountain regions and lowlands, we used the ‘Alpine Space Programme’ cooperation area, which includes the European Alps and surrounding lowlands (Supplementary Fig. 1). It extends over an area of approximately 390,000 km² and comprises Austria, Switzerland, Liechtenstein and Slovenia, as well as several regions of France, Germany and Italy. Mountain municipalities, defined here using a threshold of terrain ruggedness (difference in elevation >200 m between the value of a cell and the mean of an 8-cell neighbourhood of surrounding cells)^36^ cover 47% of the total area (37% of the municipalities) and are characterized by a high share of near-natural ecosystems, whereas lowland areas are intensively used by agriculture and industry (see Supplementary Fig. 2). Mountain municipalities are hotspots of several ecosystem services^27^, such as supplying water to downstream regions and offering a high level of recreational opportunities. Approximately 74% of the 66 million people here live in urban agglomerations in the lowland area, whereas the less populated European Alps are visited by about 120 million people each year.

Based on an exhaustive literature review, workshops with experts and a survey of users, eight key ecosystem services were identified as relevant for our study area^27^. Of these, we selected six ecosystem services to illustrate ecosystem service flows, comprising two provisioning services (fresh water, grassland biomass), two regulating services (protection against mountain hazards, carbon sequestration), and two cultural services (outdoor recreation, symbolic plants and animals).

### Ecosystem service transfer

For each ecosystem service, we identified the major direction of ecosystem service flow and the type of transfer (Fig. 1), which we supported through specific examples.

#### Fresh water

The distribution of fresh water may follow natural waterways such as rivers, but often requires a human-made carrier to transport the water from supply areas to the consumer, also defined as ‘ES commodity’^5^. The major direction of flow in this case is from the mountains to the lowlands. The selected case study focused on a water distribution company, Lake Constance Water Supply, which delivers water from Lake Constance to numerous municipalities in Baden Württemberg, Germany (https://www.bodensee-wasserversorgung.de). We quantified water supply in terms of water runoff from catchments of the Alpine space by the hydropower model from the InVEST toolbox^37^ based on root restricting layer depth (mm), plant available water content, average annual precipitation, average annual potential evapotranspiration, and land use/land cover^38^. We identified the amount of water within the watershed of Lake Constance by overlaying the water supply at the landscape scale with the boundaries of the respective watershed. To illustrate the distribution of benefiting areas, we mapped the municipalities to which the water is directly delivered as well as the pipeline system (https://www.bodensee-wasserversorgung.de). The economic value of drinking water was estimated by multiplying the volume of abstracted water with the average regional selling price of 2.15 €/m³ (https://www.statistik-bw.de).

#### Grassland biomass

Grassland biomass is partly a local service, as fodder is usually consumed where it is produced (e.g., alpine pastures). In many cases, however, demand exceeds supply and hence fodder is imported from outside. This requires transportation through human infrastructure and the direction is from global to mountain as well as lowland areas. Quantification of the supply was based on a biophysical modelling approach, where energy yields were calculated via yield functions based on the length of the growing season, precipitation during the growing season, and solar radiation^27^. The demand (amount of energy required by forage-feeding livestock) was derived from agricultural census statistics and considered herd composition, age-class energy requirements, and performance needs for milk production^27^. We summarized the supply and demand for each country in the Alpine Space (see Supplementary Tables 1 and 2). Moreover, we estimated the economic value of the produced and imported fodder applying the market price for different types of fodder in 2017 (https://www.bauernverband.de/63-betriebsmittel/futtermittel-803608, https://www.statistik.at/).

#### Protection against mountain hazards

This regulating service concentrates on avalanches, rockfalls and channel processes, and thus represents a local service restricted to mountain landscapes, meaning that no transfer occurs. Whereas other studies generally refer to the consumption of benefits where ecosystem services are generated^5,9^, in mountain areas this may also depend on the demand. We mapped supply by identifying the forest area (%) with a protective effect against potential avalanches, rockfalls and channel processes (site-protecting forest)^39^. The actual use was expressed by the forest area (%) with a protective effect for human infrastructure against potential avalanches and rockfalls (object-protecting forest)^39^. We summarized for each state in the study area the percentage of overlap between forest areas with a protective function (supply) and forest areas that effectively protect existing human settlements and infrastructure (actual use), in order to indicate the area where this service occurs (see Supplementary Table 3). For the object-protecting forest, we estimated the economic value applying the replacement cost method, implicating that there would be a societal need for protection in case the forest would be removed^40^. We used annual costs of different bioengineering technological solutions, including palisades for protection against landslides, fences for protection against avalanches and rockfall, which we multiplied with the forest area that effectively protects human settlements or infrastructure against one or more of these mountain hazards. Further details on the calculation method can be found in Supplementary Table 4 and in Häyhä *et al*.^41^.

#### Carbon sequestration

Carbon sequestration is considered a global service^5,9^. The type of ecosystem service transfer is only linked to ecological processes and can be distinguished from human-dependent types of transfer. In our study regions, there are areas that contribute to the sequestration of carbon, as well as emitting areas (demand for carbon sequestration). We assessed the supply quantifying the annual rate of CO_2_ sequestration by above- and below-ground biomass in forests based on IPCC equations^42^. To assess the demand, we used the annual rate of CO_2_ emissions based on different emissions inventories^43,44^. We then compared the amount of service demanded with the potentially supplied service at the country level for mountain and lowland areas within the study area (see Supplementary Table 5). The economic value of the supply was derived by multiplying the total amount of sequestrated carbon in the study area with the average price of emission permits in 2018 (15.89 € t^−1^, https://sandbag.org.uk/carbon-price-viewer/) traded by the European Union Emissions Trading System (EU ETS).

#### Outdoor recreation

To benefit from recreational opportunities provided by mountain environments, people living in and beyond mountain regions must travel from their place of residence to the specific mountain location, implying that people can only use this service if they can reach the locations with recreational opportunities and are allowed to access those^5^. Here we add a major direction of movement, from global to mountain areas. To analyse tourist flows to mountain regions, we selected three hotspots of actual use of outdoor recreation in the European Alps, which were identified from annual visitation rates estimated from the density of georeferenced photographs referred to user days^30^. These included the Dolomites in Italy (Bolzano, Trento and Belluno), the Northern/Central Alps in Austria (Vorarlberg, Tirol, Salzburg), a large part of the Swiss Alps (Glarus, Graubünden, Nidwalden, Obwalden, Schwyz, Uri, Ticino, Valais), and adjacent mountain ranges in northern Italy (Sondrio, Valle d’Aosta, Verbano-Cusio-Ossola). We concentrated only on regions that were completely located within the mountains, as a higher share of the social media data used for mapping referred to recreational activities in mountain areas compared to lowland areas^30^. For all regions, we collected the number of tourist arrivals in 2017 by country of origin (see Supplementary Tables 6 and 7). The local economic value was quantified based on the average daily costs and the total number of overnight stays in the respective region in 2017. Details can be found in Supplementary Table 7.

#### Symbolic plants and animals

Several plants and animals can be deemed as symbolic for the European Alps^45^, including five plants, namely edelweiss (*Leontopodium alpinum*), gentian (in particular, *Gentiana acaulis*, *Gentiana clusii*), alpenrose (*Rhododendron hirsutum*, *Rhododendron ferrugineum*), European larch (*Larix decidua*), pine (in particular, *Pinus mugo*), as well as the following five animals, Alpine ibex (*Capra ibex*), brown bear (*Ursus arctos arctos*), chamois (*Rupicapra rupicapra*), golden eagle (*Aquila chrysaetos*), marmot (*Marmota marmota*). The supply of these selected plants and animals in the European Alps was assessed based on occurrence data or by modelling their (potential) habitats; see Schirpke *et al*.^45^ for details on the mapping method.

To capture the demand for symbolic plants and animals, we used Google Trends (https://www.google.com/trends), indicating the relative popularity of the ten selected plants and animals for people worldwide. We searched for the specific names of each plant or animal in four Alpine languages (French, German, Italian and Slovenian) and additionally in English and Latin for the scientific name. For details, see Supplementary Table 8. We then collected the relative popularity scores for each plant or animal species worldwide at the country level. Finally, we calculated an average value of all ten species, applying the same weight for all species, to indicate the overall popularity of search related to symbolic plants and animals in all countries. Moreover, we assessed the number of zoos for European countries with at least one of the selected animals that are symbolic for the Alps (https://www.zootierliste.de/) as well as the number of botanical gardens that include symbolic plants from the Alps (https://www.uibk.ac.at/botany/alpine-garden/arktische-bg/index.html.en, https://www.jardinalpindulautaret.fr/).

The use of these plants and animals create among others immaterial ecosystem services (e.g., knowledge, artistic or spiritual benefits, inspiration), which are transferred from one place to another through human-made communication channels^8^. Plants and animals are often used to convey specific ideas or a certain image^45^. Accordingly, Alpine clubs of most Alpine countries refer to symbolic plants or animals in their logos: the German, Austrian and South Tyrol Alpine Clubs are represented by the edelweiss, the Swiss Alpine Club by the chamois, the Italian Alpine Club by the eagle and the Liechtenstein Alpine Club by the gentian. A continuous transfer of ideas and information additionally occurs from the Alps to people living outside the mountains, as all members regularly receive information on activities and Alpine locations via journals, newsletters, meetings, and so forth. Therefore, we mapped the number of members in these Alpine clubs at the regional level for the year 2017 (https://www.alpenverein.it; https://www.cai.it; https://www.alpenverein.de; https://www.alpenverein.li; https://www.sac-cas.ch; https://www.alpenverein.at) to illustrate the flow of information or ideas from the Alps to other regions or countries.

Another example is the airline Edelweiss Air that uses the edelweiss in its naming and logo to take a part of their home in the world (https://www.flyedelweiss.com/). We therefore mapped the number of destinations per country for the year 2019 to illustrate the flow of information or ideas from the Alps, in this specific case from Switzerland, to other countries (https://www.flyedelweiss.com/).

## Supplementary information

Supplementary Information

## Data Availability

All data analysed in this study are publicly available. Data on ecosystem services at the municipality level are available from www.alpes-webgis.eu. Data summarized at the national level and related data sources are reported in Supplementary Tables 1–9.
