# Supplementary material for: A transnational perspective of global and regional ecosystem service flows from and to mountain regions
Source: Sci Rep. 2019 Apr 30;9:6678. doi: 10.1038/s41598-019-43229-z (PMC6491654; doi:10.1038/s41598-019-43229-z)
Supplement: Supplementary file 1 — Supplementary Information [file 41598_2019_43229_MOESM1_ESM.pdf]

A transnational perspective of global and regional ecosystem service flows from and to mountain regions

Uta Schirpke, Ulrike Tappeiner, Erich Tasser

Supplementary Information

Content

Supplementary Fig. 1. Location of the Alpine Space study area 3

Supplementary Fig. 2. Distribution of major land cover types based on Corine land cover 4

Supplementary Table 1. Supply and demand of fodder within the study area 5

Supplementary Table 2. Distribution of imported fodder 6

Supplementary Table 3. Distribution of forest area with different functions referred to the total forest area 7

Supplementary Table 4. Economic valuation of mountain hazards 8

Supplementary Table 5. Supply of and demand for carbon sequestration 9

Supplementary Table 6. Most important countries of origin of visitors in 2017 to major hotspots of outdoor recreation in the European Alps 10

Supplementary Table 7. Origin of visitors, number of overnight stays and local economic value of three tourism hotspots in the European Alps (year 2017) 11

Supplementary Table 8. Terms used in Google Trends queries for symbolic plants and animals 12

Supplementary Table 9. Popularity of search terms for symbolic plants and animals........ 13

Supplementary references 15

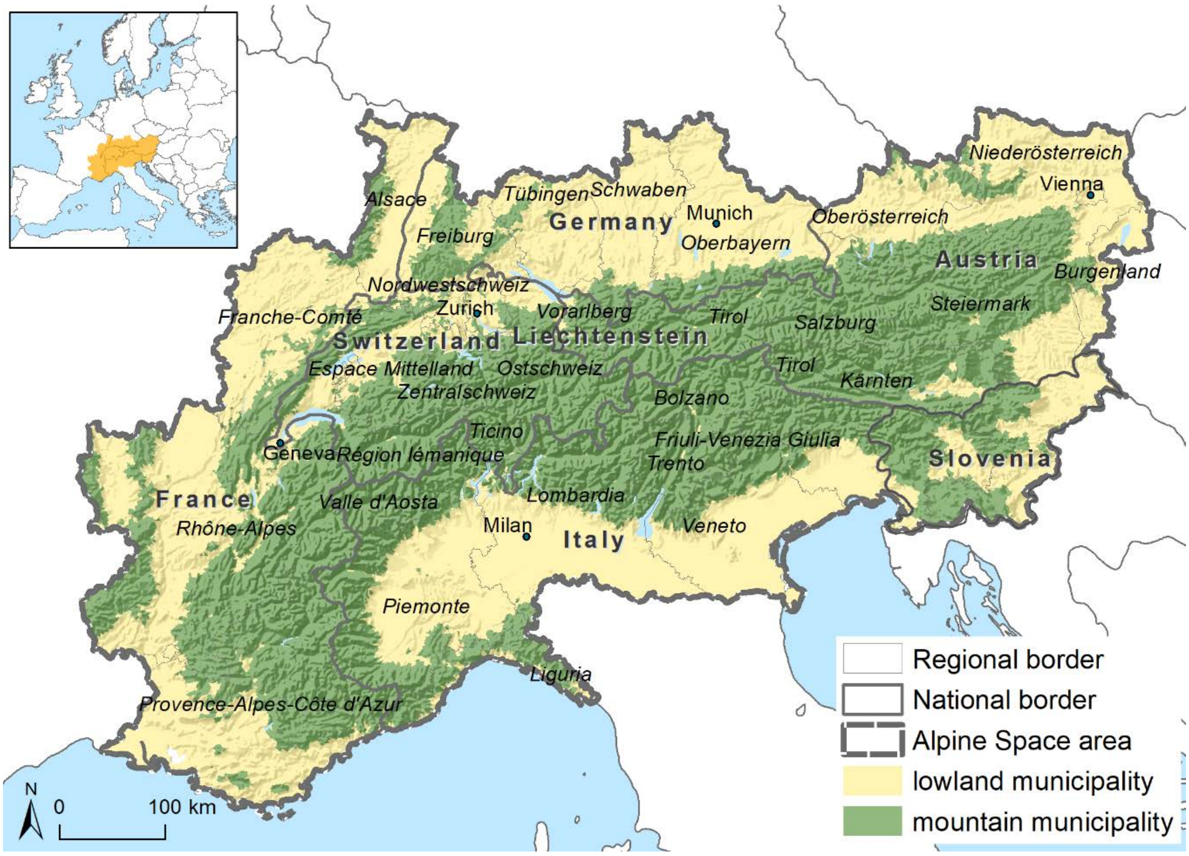

Supplementary Fig. 1. Location of the Alpine Space study area

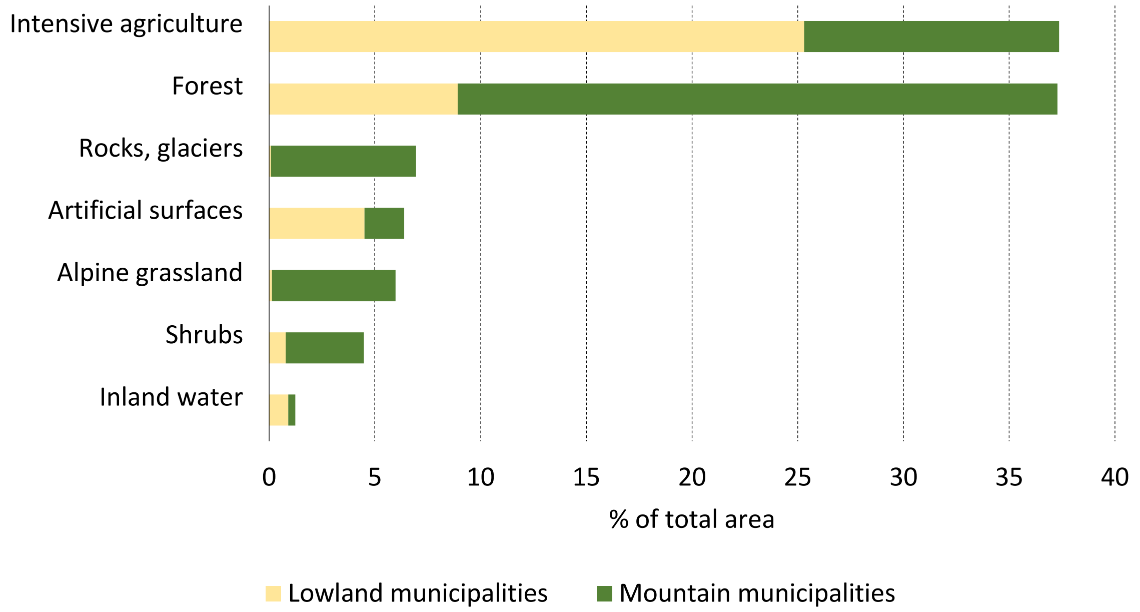

Supplementary Fig. 2. Distribution of major land cover types based on Corine land cover 2012

Data source: http://land.copernicus.eu/pan-european/corine-land-cover/clc-2012

Supplementary Table 1. Supply and demand of fodder within the study area

Country	Supply (MJ ME y^{1})	Demand (MJ ME y^{-1})	Produced fodder (million € y^{-1})	Imported fodder (million € y^{-1})
Austria	143,127,000,000	139,446,067,886	1,520.8	318.2
France	119,826,000,000	112,067,776,680	1,312.9	148.9
Germany	158,280,000,000	128,587,835,327	1,737.6	-102.7
Italy	83,154,184,947	316,447,766,408	912.6	3,726.5
Lichtenstein	77,488,032	357,790,333	0.7	4.6
Slovenia	19,514,436,649	31,832,165,503	205.5	240.4
Switzerland	63,542,110,415	103,280,290,662	656.9	791.4

Data sources: [1-3], www.alpes-webgis.eu, https://www.ble.de, https://www.ovid-verband.de/, https://www.bauernverband.de/63-betriebsmittel/futtermittel-803608, https://www.statistik.at/

Supplementary Table 2. Distribution of imported fodder

Country	Imported metabolizable energy (% of total energy import)
Argentina	6.5
Australia	0.8
Austria	0.0
Belgium	1.2
Brazil	14.0
Bulgaria	0.3
Canada	0.8
Cyprus	0.1
Czech Republic	1.7
Denmark	1.8
Estonia	0.6
Finland	1.9
France	16.9
Germany	8.9
Greece	0.8
Hungary	0.9
Ireland	2.0
Italy	4.4
Kazakhstan, Ukraine	0.8
Latvia	1.1
Lithuania	1.2
Luxembourg	0.1
Malta	0.0
Netherlands	1.8
Norway	0.8
Paraguay	1.6
Poland	3.5
Portugal	1.3
Romania	2.4
Russia	0.1
Slovakia	0.9
Slovenia	0.0
Spain	2.3
Sweden	3.3
Switzerland	0.0
United Kingdom	4.3
United States	4.5
Uruguay	0.6

Data sources [1, 2], https://www.ble.de, https://www.ovid-verband.de/

Supplementary Table 3. Distribution of forest area with different functions referred to the total forest area

Country	Site-protection forest (%)	Site- and object-protection forest (%)	Remaining forest (%)	Total forest area (km^2)
Austria	15	4	81	382.56
France	7	3	90	417.49
Germany	5	1	93	165.28
Italy	18	7	75	386.69
Liechtenstein	18	11	71	0.69
Slovenia	8	2	90	128.07
Switzerland	9	7	84	127.70

Data sources: www.alpes-webgis.eu

Supplementary Table 4. Economic valuation of mountain hazards

Type of mountain hazard	Forest area (ha)	Bioengineering technology	Cost (€ ha^{-1} y^{-1})	Source	Economic value (Mio € y^{-1})
Avalanche release area	406,968	Snow fence	3,675	[4]	1,495.61
Rockfall path	395,914	Rockfall fence	2,771	own calculation based on [4, 5]	1,096.98
Avalanche release area + rockfall path	42,872	Snow fence + rockfall fence	6,446	own calculation based on [4, 5]	276.34
Debris flow buffer	2,055,928	Simple palisade	1,129	[4]	2,321.14
**Total annual economic value (Mio € y^{-1})**					**5,190.08**

Supplementary Table 5. Supply of and demand for carbon sequestration

Country	Lowland municipalities		Mountain municipalities		Economic value of sequestrated carbon (€ y^{-1})
	Supply	Demand	Supply	Demand	
	(t CO_2 y^{-1})				
Austria	2291	18434	5290	6603	120,469
France	11073	175073	14237	89627	402,188
Germany	2947	18321	1853	3094	76,279
Italy	3322	153222	10498	57530	219,605
Liechtenstein	2	4	44	25	729
Slovenia	417	12203	922	1157	21,280
Switzerland	2258	20445	5749	18405	127,238

Data sources: supply [6], demand [7, 8]

Supplementary Table 6. Most important countries of origin of visitors in 2017 to major hotspots of outdoor recreation in the European Alps

Country	Arrivals (n y^{-1})	Country	Arrivals (n y^{-1})
Argentina	13,161	Luxembourg	88,859
Australia	132,457	Malaysia	6,171
Austria	4,470,500	Malta	12,247
Bahrain	1,266	Mexico	7,797
Belgium	683,455	Netherlands	1,916,152
Brazil	59,460	New Zealand	18,284
Bulgaria	36,975	Norway	111,758
Belarus	1,823	Oman	1,459
Canada	81,436	Philippines	1,413
Chile	1,187	Poland	448,426
China	707,777	Portugal	29,846
Croatia	64,162	Qatar	2,553
Cyprus	3,989	Romania	134,647
Czech Republic	666,957	Russia	232,867
Denmark	376,577	Saudi Arabia	88,008
Egypt	2,353	Serbia	1,559
Estonia	22,025	Singapore	9,465
Finland	92,159	Slovakia	29,673
France	712,836	Slovenia	108,970
Germany	14,254,195	South Africa	19,055
Greece	24,996	South Korea	177,833
Hungary	186,361	Spain	201,413
Iceland	14,229	Sweden	285,878
India	179,650	Switzerland	5,201,355
Indonesia	7,359	Taiwan	95,278
Ireland	74,583	Thailand	29,600
Israel	153,866	Turkey	33,841
Italy	7,647,821	Ukraine	40,036
Japan	147,542	United Arab Emirates	78,530
Kuwait	4,108	United Kingdom	954,627
Latvia	26,223	United States	575,662
Liechtenstein	5,517	Venezuela	1,265
Lithuania	33,965		

Data sources:
https://www.statistik.at/web_de/statistiken/wirtschaft/tourismus/beherbergung/ankuenfte_naechtigungen/index.html, https://www.bfs.admin.ch/bfs/en/home/statistics/tourism/surveys/hesta.html, https://www.istat.it/

Supplementary Table 7. Origin of visitors, number of overnight stays and local economic value of three tourism hotspots in the European Alps (year 2017)

	Country	Dolomites	Northern Central Alps	Swiss/Italian Alps
Origin of visitors	Germany	32.6	44.6	9.3
	Italy	45.8	2.4	19.1
	Switzerland	3.5	5.2	43.8
	Austria	3.7	18.4	0.9
	Netherlands	2.0	7.1	2.0
	Other European countries	11.2	17.0	17.0
	Non-European countries	1.1	5.4	7.9
Number of overnight stays (persons y^{-1})		83,806,251	85,136,794	13,679,928
Local economic value (billion € y^{-1})		6.62	6.83	2.60

Data sources:
https://www.statistik.at/web_de/statistiken/wirtschaft/tourismus/beherbergung/ankuenfte_naechtigungen/index.html, https://www.bfs.admin.ch/bfs/en/home/statistics/tourism/surveys/hesta.html, https://www.istat.it/, https://de.statista.com/statistik/daten/studie/252336/umfrage/reisedauer-und-reisekosten-der-deutschen/

Supplementary Table 8. Terms used in Google Trends queries for symbolic plants and animals (https://www.google.com/trends). The terms in different languages were linked with + (corresponds to or) for each plant or animal species.

English	French	German	Italian	Slovenian	Latin
Alpine chamois	chamois	Gämse	camoscio	gams1	Rupicapra rupicapra
Alpine marmot	marmotte	Alpenmurmeltier	marmotta	svizec	Marmota marmota
Alpine ibex	capricorne	Alpensteinbock	capricorno	kozorog	Capra ibex
European brown bear	ours	Braunbär	orso	rjavi	Ursus arctos arctos
European golden eagle	aigle royal	Steinadler	aquila reale	planinski orel	Aquila chrysaetos
Alpine rhododendron	alpenrose	Alpenrose	rododendro	sleč	Rhododendron
Alpine edelweiss	edelweiss	Edelweiß	stella alpina	planika	Leontopodium alpinum
Alpine gentian	gentiane	Enzian	genziana	encijan	Gentiana
European larch	mélèze	Europäische Lärche	larice	macesen	Larix decidua
Alpine pine	pin des montagnes	Latschenkiefer	pino mugo	ruševje	Pinus mugo

[1] Term not used because of no comparable overlap with terms from other languages.

Supplementary Table 9. Popularity of search terms for symbolic plants and animals.
The inserted search queries that were used in Google Trends (https://www.google.com/trends) are reported in Supplementary Table 7.

Country	Chamois	Marmot	Alpine ibex	Brown bear	Golden eagle	Alpenrose	Edelweiss	Gentian	Larch	Pine	Mean value
Albania	0.0	0.0	0.0	20.3	0.0	0.0	0.8	78.8	0.0	0.0	11.8
Algeria	35.7	35.7	0.0	0.0	28.6	0.0	0.0	0.0	0.0	0.0	2.8
Argentina	14.3	0.0	0.0	14.3	0.0	28.6	42.9	0.0	0.0	0.0	0.7
Australia	43.2	5.4	0.0	2.7	0.0	35.1	5.4	8.1	0.0	0.0	3.7
Austria	7.4	1.6	0.8	0.3	26.3	20.3	6.6	23.2	0.0	13.7	38.0
Bangladesh	0.0	0.0	0.0	0.0	0.0	100.0	0.0	0.0	0.0	0.0	0.2
Belarus	0.0	0.0	0.0	0.0	0.0	0.0	100.0	0.0	0.0	0.0	0.1
Belgium	11.0	38.7	0.0	0.6	4.4	16.6	3.9	10.5	3.3	11.0	18.1
Bosnia and Herzegovina	0.0	0.0	0.0	0.0	0.0	6.4	93.6	0.0	0.0	0.0	4.7
Brazil	40.0	5.0	5.0	10.0	0.0	10.0	10.0	10.0	10.0	0.0	1.0
Bulgaria	0.0	0.0	0.0	0.0	0.0	42.9	57.1	0.0	0.0	0.0	0.7
Canada	24.4	32.2	0.0	0.5	4.9	12.7	4.9	4.9	2.9	12.7	10.3
Chile	0.0	0.0	0.0	0.0	0.0	66.7	33.3	0.0	0.0	0.0	0.9
China	0.0	0.0	0.0	45.5	0.0	27.3	27.3	0.0	0.0	0.0	1.1
Colombia	87.2	0.0	0.0	5.1	0.0	5.1	2.6	0.0	0.0	0.0	2.0
Costa Rica	0.0	0.0	0.0	0.0	0.0	0.0	100.0	0.0	0.0	0.0	0.3
Croatia	0.0	0.0	11.8	3.9	0.0	15.7	15.7	52.9	0.0	0.0	5.1
Cuba	0.0	0.0	0.0	0.0	0.0	0.0	100.0	0.0	0.0	0.0	0.7
Cyprus	0.0	0.0	0.0	0.0	0.0	0.0	100.0	0.0	0.0	0.0	0.7
Czech Republic	0.0	0.0	0.0	0.8	0.0	15.4	2.4	17.1	0.0	64.2	12.3
Denmark	7.4	39.0	0.0	0.7	0.0	44.9	2.2	5.9	0.0	0.0	13.6
Dominican Republic	0.0	0.0	0.0	0.0	0.0	0.0	100.0	0.0	0.0	0.0	0.2
Egypt	66.7	0.0	0.0	11.1	0.0	0.0	22.2	0.0	0.0	0.0	0.5
Estonia	0.0	0.0	0.0	0.0	0.0	0.0	100.0	0.0	0.0	0.0	0.7
Finland	14.3	0.0	0.0	4.8	0.0	47.6	9.5	23.8	0.0	0.0	2.1
France	28.3	35.8	0.4	0.4	5.4	7.5	1.8	11.8	3.2	5.4	27.9
Georgia	0.0	0.0	0.0	0.0	0.0	0.0	100.0	0.0	0.0	0.0	0.2
Germany	6.9	2.3	0.6	0.6	6.9	31.6	5.7	24.1	0.6	20.7	17.4
Greece	0.0	0.0	0.0	16.7	0.0	33.3	50.0	0.0	0.0	0.0	0.6
Hungary	0.0	0.0	0.0	7.1	0.0	30.4	5.4	0.0	0.0	57.1	5.6
India	13.6	0.0	2.3	2.3	0.0	13.6	50.0	4.5	0.0	13.6	2.2
Indonesia	44.4	0.0	0.0	5.6	0.0	11.1	33.3	5.6	0.0	0.0	0.9
Ireland	35.7	0.0	0.0	7.1	0.0	50.0	7.1	0.0	0.0	0.0	2.8
Israel	0.0	0.0	0.0	0.0	0.0	0.0	100.0	0.0	0.0	0.0	0.1
Italy	15.9	12.9	15.9	15.9	3.7	3.3	1.9	8.3	6.5	15.9	63.0
Japan	28.6	0.0	0.0	14.3	0.0	14.3	14.3	28.6	0.0	0.0	0.4
Kazakhstan	0.0	0.0	0.0	0.0	0.0	0.0	100.0	0.0	0.0	0.0	0.1
Latvia	0.0	0.0	0.0	0.0	0.0	83.3	16.7	0.0	0.0	0.0	1.2
Liechtenstein	0.0	0.0	0.0	0.0	0.0	0.0	100.0	0.0	0.0	0.0	10.0
Lithuania	0.0	0.0	0.0	0.0	0.0	88.9	11.1	0.0	0.0	0.0	0.9
Luxembourg	0.0	0.0	0.0	0.0	0.0	78.2	21.8	0.0	0.0	0.0	5.5
Macedonia	0.0	0.0	0.0	0.0	0.0	0.0	100.0	0.0	0.0	0.0	1.0
Malaysia	50.0	0.0	0.0	0.0	0.0	33.3	16.7	0.0	0.0	0.0	0.6
Maldives	0.0	0.0	0.0	0.0	0.0	0.0	100.0	0.0	0.0	0.0	1.1
Morocco	40.0	56.0	0.0	0.0	0.0	0.0	4.0	0.0	0.0	0.0	2.5
Mauritius	0.0	0.0	0.0	0.0	0.0	0.0	100.0	0.0	0.0	0.0	0.7
Mexico	28.6	0.0	0.0	14.3	0.0	28.6	28.6	0.0	0.0	0.0	0.4
Moldova	0.0	0.0	0.0	0.0	0.0	0.0	100.0	0.0	0.0	0.0	0.5
Montenegro	0.0	0.0	0.0	0.0	0.0	0.0	100.0	0.0	0.0	0.0	1.9
Nepal	0.0	0.0	0.0	0.0	0.0	100.0	0.0	0.0	0.0	0.0	5.0

Country	Chamois	Marmot	Alpine ibex	Brown bear	Golden eagle	Alpenrose	Edelweiss	Gentian	Larch	Pine	Mean value
Netherlands	6.6	32.9	0.0	0.5	0.0	27.2	5.6	10.3	0.0	16.9	10.7
New Zealand	39.7	0.0	0.0	0.0	0.0	56.9	3.4	0.0	0.0	0.0	5.8
Norway	20.5	0.0	0.0	0.0	0.0	72.7	6.8	0.0	0.0	0.0	4.4
Pakistan	0.0	0.0	0.0	0.0	0.0	66.7	33.3	0.0	0.0	0.0	0.2
Paraguay	0.0	0.0	0.0	0.0	0.0	0.0	100.0	0.0	0.0	0.0	0.4
Peru	0.0	0.0	0.0	0.0	0.0	0.0	100.0	0.0	0.0	0.0	0.1
Philippines	81.8	0.0	0.0	0.0	0.0	9.1	9.1	0.0	0.0	0.0	1.1
Poland	5.6	1.9	0.0	1.9	0.0	13.0	1.9	7.4	0.0	68.5	5.4
Portugal	0.0	0.0	0.0	7.7	0.0	61.5	30.8	0.0	0.0	0.0	0.7
Romania	4.1	0.0	0.0	3.1	0.0	9.3	5.2	70.1	8.2	0.0	9.7
Russia	8.0	0.0	0.0	4.0	0.0	8.0	8.0	8.0	0.0	64.0	1.3
Saudi Arabia	0.0	0.0	0.0	0.0	0.0	0.0	100.0	0.0	0.0	0.0	0.1
Serbia	0.0	0.0	0.0	0.0	0.0	10.8	29.7	59.5	0.0	0.0	3.7
Singapore	46.7	0.0	0.0	0.0	0.0	20.0	33.3	0.0	0.0	0.0	1.5
Slovenia	0.0	17.5	14.6	4.0	3.1	2.7	4.3	9.7	22.5	21.6	49.4
Slovakia	0.0	0.0	0.0	0.0	0.0	10.4	3.1	11.5	0.0	75.0	9.6
South Africa	61.5	0.0	0.0	0.0	0.0	15.4	23.1	0.0	0.0	0.0	1.3
South Korea	0.0	0.0	0.0	0.0	0.0	66.7	33.3	0.0	0.0	0.0	0.3
Spain	10.7	17.9	3.6	3.6	0.0	17.9	14.3	7.1	0.0	25.0	2.8
Sri Lanka	0.0	0.0	0.0	0.0	0.0	0.0	100.0	0.0	0.0	0.0	0.2
St. Helena	0.0	0.0	0.0	0.0	0.0	0.0	100.0	0.0	0.0	0.0	0.4
Sweden	12.0	6.8	0.0	0.9	0.0	54.7	6.8	18.8	0.0	0.0	5.9
Switzerland	13.8	10.6	1.3	1.1	5.5	18.3	17.1	18.3	2.8	11.2	54.5
Taiwan	66.7	0.0	0.0	0.0	0.0	16.7	16.7	0.0	0.0	0.0	0.6
Thailand	44.4	0.0	0.0	11.1	0.0	22.2	22.2	0.0	0.0	0.0	0.5
Turkey	25.0	0.0	0.0	12.5	0.0	25.0	12.5	25.0	0.0	0.0	0.4
Ukraine	0.0	0.0	0.0	11.1	0.0	22.2	22.2	44.4	0.0	0.0	0.5
United Arab Emirates	60.0	0.0	0.0	0.0	0.0	20.0	20.0	0.0	0.0	0.0	1.0
United Kingdom	30.1	14.2	1.8	1.8	0.9	24.8	3.5	5.3	0.0	17.7	5.7
United States	21.2	1.9	1.0	1.9	0.0	25.0	5.8	13.5	1.0	28.8	5.2
Vietnam	0.0	0.0	20.0	20.0	0.0	20.0	20.0	20.0	0.0	0.0	0.3

Supplementary references

1. Huyghe, C., De Vliegher, A., Van Gils, B. & Peeters, A. in *Grasslands and herbivore production in Europe and effects of common policies* (Editions Quae, 2014).
2. Wedlich, K. V., Franzaring, J. & Fangmeier, A. in *Entwicklung und Erprobung eines Konzepts für ein Monitoring von für den Import zugelassenem transgenem Raps nach Richtlinie 2001/18/EG: Ergebniss eines F E-Vorhabens (FKZ 3511 89 0100) des Bundesamtes für Naturschutz* (Bundesamt für Naturschutz, 2016).
3. Schirpke, U. *et al*. Integrating supply, flow and demand to enhance the understanding of interactions among multiple ecosystem services. *Sci Total Environ* **651**, 928-941 (2019).
4. Häyhä, T., Franzese, P. P., Paletto, A. & Fath, B. D. Assessing, valuing, and mapping ecosystem services in Alpine forests. *Ecosystem Services* **14**, 12-23 (2015).
5. Bundesministerium für Land- und Forstwirtschaft, Umwelt und Wasserwirtschaft. *Rock 'n' Roll am Berghang - Steinschlagschutz in Österreich*. BMLFUW, UW-Nr. 907, Wien. (2015).
6. Eggleston, H., Buendia, L., Miwa, K., Ngara, T. & Tanabe, K. 2006 IPCC guidelines for national greenhouse gas inventories. *A report prepared by the Task Force on National Greenhouse Gas Inventories of the Intergovernmental Panel on Climate Change.(Institute for Global Environmental Strategies: Hayama, Japan) Available at:(accessed 2 April 2008)* (2006).
7. Trombetti, M., Pisoni, E. & Lavalle, C. *Downscaling methodology to produce a high resolution gridded emission inventory to support local/city level air quality policies*. Office for Official Publications of the European Communities, Luxembourg EUR **28428** (2017).
8. European Commission. Emission Database for Global Atmospheric Research (EDGAR), release EDGARv4.2 FT2012. (2014).
